# Correlation between ultrasound velocity and densitometry in fresh and demineralized cortical bone

**DOI:** 10.6061/clinics/2016(11)07

**Published:** 2016-11

**Authors:** Alessandro Queiroz de Mesquita, Giuliano Barbieri, Claudio Henrique Barbieri

**Affiliations:** Faculdade de Medicina de Ribeirão Preto da Universidade de São Paulo, Departamento de Biomecânica, Medicina e Reabilitação do Aparelho Locomotor, Ribeirão Preto/SP, Brazil

**Keywords:** Bone, Demineralization, Ultrasound Velocity, Bone Mineral Density

## Abstract

**OBJECTIVE:**

To compare ultrasound propagation velocity with densitometry in the diaphyseal compact cortical bone of whole sheep metatarsals.

**METHODS:**

The transverse ultrasound velocity and bone mineral density of 5-cm-long diaphyseal bone segments were first measured. The bone segments were then divided into four groups of 15 segments each and demineralized in an aqueous 0.5 N hydrochloric acid solution for 6, 12, 24 or 36 hours. All measurements were repeated after demineralization for each time duration and the values measured before and after demineralization were compared.

**RESULTS:**

Ultrasound velocity and bone mineral density decreased with demineralization time, and most differences in the pre- and post-demineralization values within each group and between groups were significant: A moderate correlation coefficient (r=0.75956) together with a moderate agreement was determined between both post-demineralization parameters, detected by the Bland-Altman method.

**CONCLUSION:**

We conclude that both ultrasound velocity and bone mineral density decrease as a result of demineralization, thus indicating that bone mineral content is of great importance for maintaining the acoustic parameters of cortical bone, as observed for cancellous bone. Ultrasound velocity can be used to evaluate both compact cortical bone quality and bone mineral density.

## INTRODUCTION

Bone tissue is a composite material that primarily consists of collagen fibers and an inorganic matrix. The mechanical properties of bone vary according to site and direction (anisotropy) and also depend on the relative proportion of each component at different sites. The mineral component of bone accounts for approximately 65% of its dry mass, most of which is hydroxyapatite [Ca10(PO4)6(OH)2]. As hydroxyapatite is a very prevalent component in bone, it plays an important role in ultrasound transmission through the bone, which has been well established in the literature.

Dual-energy x-ray absorptiometry (DEXA), commonly known as densitometry, is the tool most frequently used to assess bone mass. DEXA is based on a low radiation dose (1-6 µSv) and can rapidly obtain measurements (∼2 min), although the method is size-dependent and provides an areal density (g/cm2) rather than a volumetric density (g/cm3) 1. Nevertheless, it is currently the gold standard for diagnosing osteoporosis and assessing fracture risk.

The quantitative ultrasonic properties (ultrasound absorption, velocity and attenuation) of any biological tissue are primarily determined by protein constituents at the macromolecular level, particularly collagen. The ultrasound velocity is significantly higher in collagen-rich tissues 2,3,4, the ultrasonic properties of which are also affected by the direction ultrasound is applied (angular dependence) 5.

Ultrasound transmission and propagation vary according to the local bone composition and the direction of wave insertion, although quantitative ultrasound, or ultrasonometry, can still provide a very distinct measure of bone anisotropy, thus providing an indirect measure of bone quality and mechanical properties that is somewhat comparable to densitometry 6,7,8,9,10. The correlation between ultrasound velocity (UV) and attenuation and volumetric density (Archimedes principle) was established for cancellous bone approximately 25 years ago, and UV was found to be the most sensitive parameter 11. In a more recent and sophisticated study using ultrasonometry associated with Fourier analysis, UV, elasticity and density were compared in cancellous bone specimens, and good correlation was found between UV and density but not between UV and elasticity 12.

The ability of ultrasound to predict the mechanical properties of bone with varying levels of mineral content was confirmed by comparing bone elastic moduli (biomechanical essays) with acoustic impedance results, and a strong positive correlation was demonstrated between the two parameters. This indicated that the acoustic impedance was a strong predictive factor of the elastic modulus and can help predict the mechanical properties of bone tissue as a function of mineral content 13.

The correlation between UV and bone mineral density (BMD) has also been demonstrated in clinical studies, which are more attractive to clinicians who frequently interact with patients. Some clinical studies have been conducted to compare UV with BMD as measured by DEXA at different sites of normal 14,15 and pagetic bone 16. These studies have shown a positive correlation between UV and BMD, therefore suggesting that UV is an accurate method for assessing osteoporosis.

The mechanical properties, BMD and UV, and broadband ultrasound attenuation (BUA) were also compared in human and bovine bone, leading to the conclusion that BMD and UV were the most accurate parameters for predicting the mechanical properties of high density trabecular bone 17,18.

According to the above-mentioned references, most research in the field of bone ultrasonometry has considered trabecular bone, whereas few studies have considered compact cortical diaphyseal bone, and hardly any studies have evaluated whole cylindrical diaphyseal bone. We hypothesized that the ultrasonic behavior of cortical bone would be somewhat similar to that of trabecular bone and that a reduction in BMD would therefore imply a reduction in the ultrasonic properties of bone, even whole diaphyseal bone. To test this hypothesis, we measured UV and BMD before and after demineralization of the whole metatarsal bone of sheep. Both UV and BMD were measured using the appropriate specific equipment.

## MATERIAL AND METHODS

This experiment was approved by the Ethics Committee on the Experimental Use of Animals of the authors’ institution (Protocol #035/2010). Sixty fresh sheep metatarsals (Santa Ignez breed, male, 15 months of age on average, for human consumption) were obtained from a local authorized slaughterhouse.

**Figure 1:**
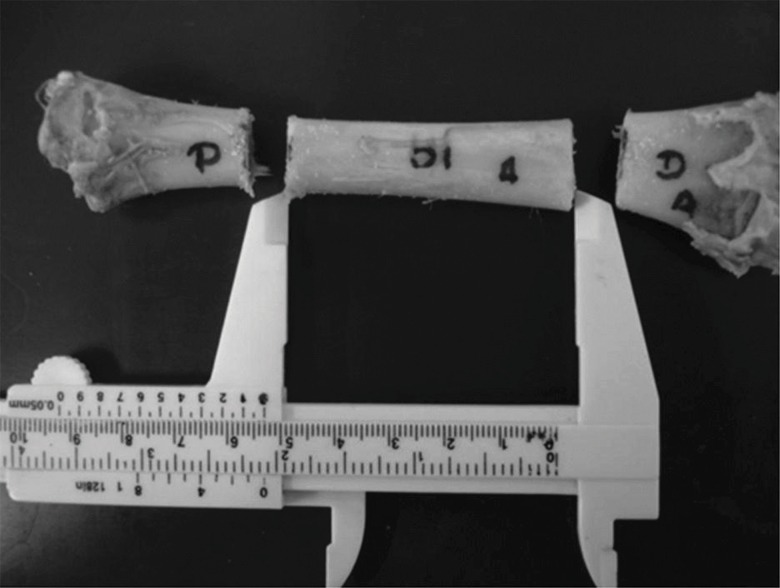
Photograph of a 6-cm-long diaphyseal bone segment that was obtained.

All bones were completely freed from any soft tissue, including the periosteum, by careful dissection and stripping, and divided into three segments of equal length (∼5 cm). Only the middle segment was used for the analyses because we were particularly interested in cortical bone (Figure 1). The bone segments were initially identified, weighed, radiographed and divided into four groups (G6, G12, G24 and G36) of 15 specimens each according to demineralization time. They were then submitted to pre-demineralization UV and BMD analyses, and all parts were thereafter decalcified. Conventional radiographs were recorded and UV and BMD measurements were repeated after demineralization and pre- and post-demineralization images and figures were compared.

### Bone demineralization procedure

**Figure 2:**
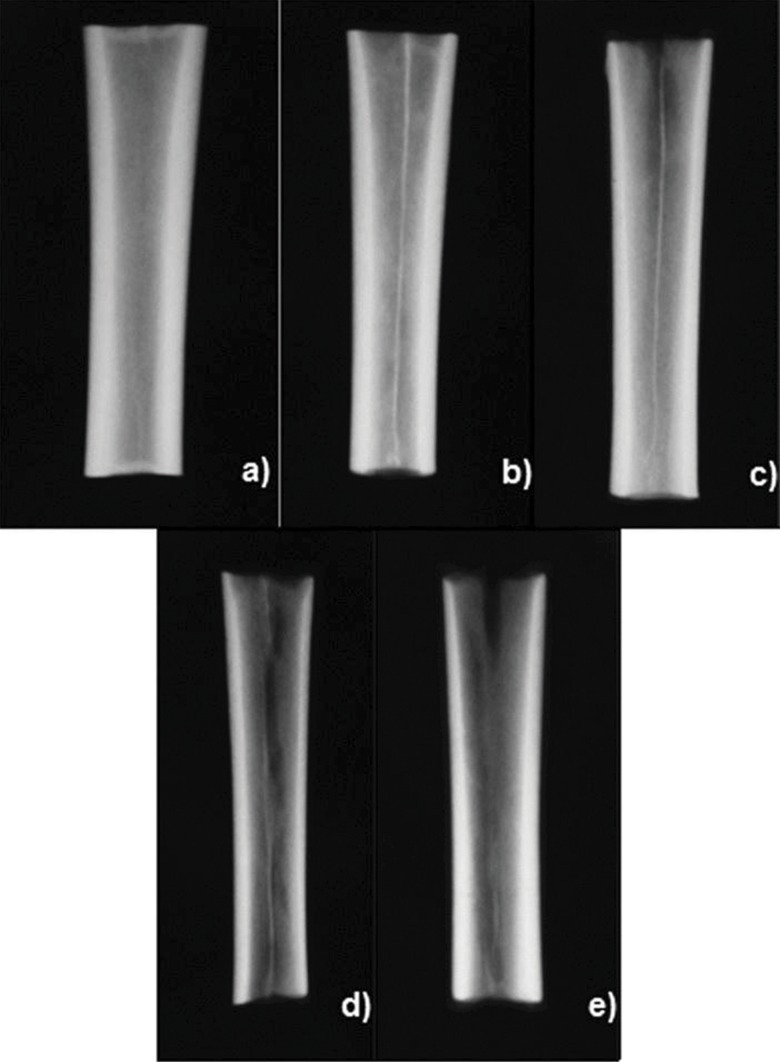
Radiographic appearance of the bone segments after the demineralization process: before demineralization (a) and after 6 (b), 12 (c), 24 (d) and 36 (e) hours. Differences are not apparent, except for some thinning of the cortex and rarefaction of the medullary cancellous bone.

The bone segments were individually measured for volume via water column dislocation 19 and then placed in identified glass flasks containing 20 times the segment’s volume of an aqueous 0.5 N hydrochloric acid solution. The segments were left to stand for 6, 12, 24 or 36 hours according to the group. After the demineralization period, the bone segments were thoroughly washed with deionized distilled water and again submitted to UV and BMD measurements and radiography (Figure 2).

### Ultrasonometry

**Figure 3:**
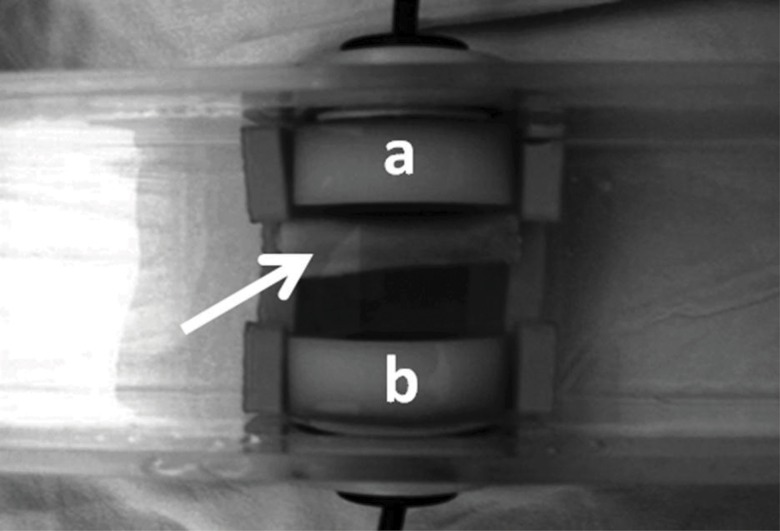
The diaphyseal segment inside the acoustic tank (arrow) positioned between the transmitting (a) and receiving (b) ultrasound transducers.

Transverse underwater UV was measured with the bone segments immersed in an acoustic tank that was adapted with two unfocused ultrasound transducers (2 mm-thick PZT-5 disc, 12 mm in diameter, 1 MHz frequency), one on each of the tank in the geometric center of the walls and separated by a 25 mm distance but precisely aligned with each other along the central longitudinal axis (Figure 3). To record measurements, the two transducers, one for US wave emission and the other for reception, were connected to an ultrasound generator-receiver amplifier source (Biotecnosis do Brasil Ltda., Model US01, Ribeirão Preto, São Paulo, Brazil, www.biotecnosis.com) that could generate high power (up to 300 V) narrow (1 µs) well-defined ultrasonic pulses. The source was linked to a computer loaded with software to automatically calculate the UV based on the time of flight of the first arrived signal (FAS), as observed on a digital storage oscilloscope (Agilent Technologies, Inc., model DSO3062A, Shangai, China), together with the emitted ultrasound signal.

**Figure 4:**
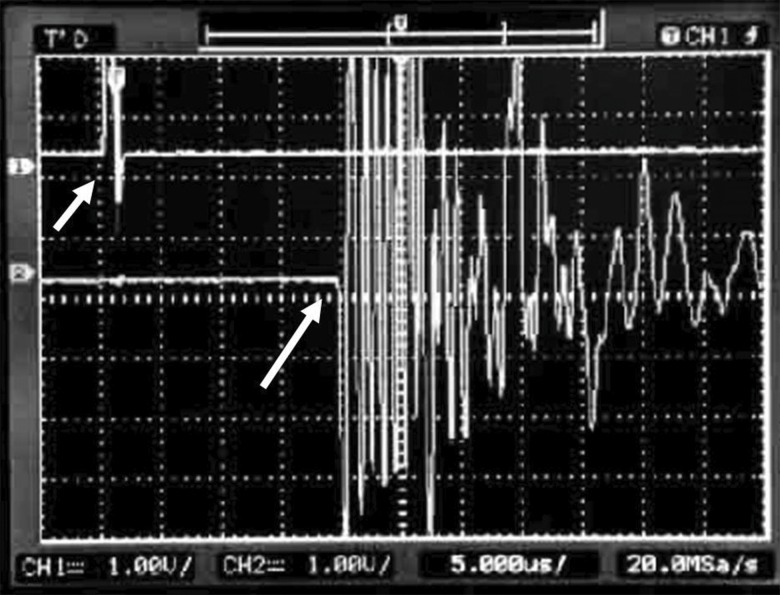
The emitted US wave (short arrow) and the first arrived US signal (FAS, long arrow), as observed using an oscilloscope.

The FAS was defined as the first 5% positive deflection above baseline preceding the signal corresponding to the transmission mean (water) (Figure 4). The equipment was calibrated after every five measurements with a 20-mm-thick Teflon disc (constant 1274 m/s UV, at 35°C, 0.3% variation) to check for regularity and accuracy. The bone segments were positioned lengthwise in the acoustic tank supported by a Teflon stand on each end, transverse to the transducers and with the midpoint of the bone segment precisely aligned with the central axis of both transducers but closer to the emitting transducer than to the receiving transducer. The ultrasound emission frequency was 1 MHz, the pulse duration was ±1 μs, the rise time was ±0.1 ns, and the repetition time was 1 s. UV was calculated according to the formula:




(1)

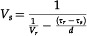




where *V_s_*=velocity through the specimen; *V_r_*=velocity through the reference propagation medium (water); *τ_r_*=time for reference propagation medium alone (water); *τ_s_*=time for reference propagation medium and specimen; and *d*</emph>=distance (coronal diameter of each individual bone segment, corresponding to the distance travelled by the ultrasound waves through the bone).

Five consecutive measurements were made at 5-minute intervals, the lowest and highest values were discarded, and the mean of the three remaining values was used for statistical comparisons.

### Densitometry

BMD was measured by DEXA in each individual segment using a clinical densitometer (Hologic Inc., model QDR4500 Elite, Bedford, MA, USA, www.hologic.com). Each tibial segment was positioned lengthwise directly on the table without any other material and precisely centered below the beam with the aid of the laser collimator. Three consecutive measurements were made, and the mean value was calculated and used for statistical comparisons. The results are reported as g/cm^2^.

Although a regularly checked densitometer currently used for clinical purposes was employed, the precision error was calculated for the untouched bone segments (before demineralization) according to the procedure recommended by the International Society for Clinical Densitometry (ISCD) and adopted by the densitometry facility of our institution. The calculated precision error was 1.9% with a least significant change (LSC) of 5.2%.

### Statistical analysis

Two-way analysis of variance (ANOVA) was used to compare the individual results of UV and BMD before and after demineralization 20. Data referring to UV and BMD before and after demineralization were compared using a mixed effects linear model, adjusted by the Proc Mixed procedure of SAS v.9 software at a 5% significance level (*p*≤0.05). A raw comparison between average values was also undertaken for each situation and the Pearson correlation coefficient was proposed to quantify the correlation between UV and BMD. Bland-Altman graphs were used to evaluate the agreement between the two previous methods 21.

## RESULTS

The radiographic appearance of the specimens did not substantially change with the time of demineralization, but some thinning of the cortical layer, dark spots and areas of more pronounced demineralization were visible (Figure 2).

### Ultrasound velocity

**Figure 5:**
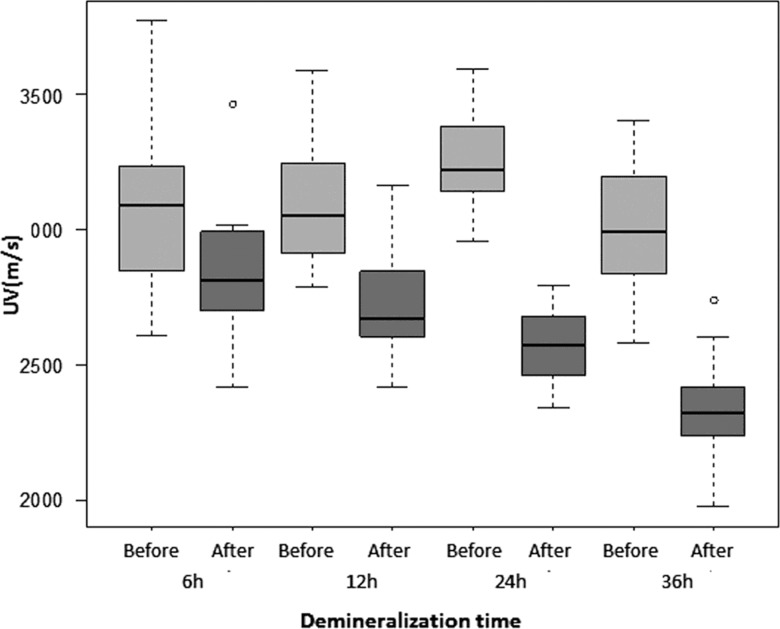
Box-plot graph of UV before (light grey boxes) and after (dark grey boxes) demineralization, according to demineralization time.

The pre-demineralization UV, as measured separately for each group (G6, G12, G24 and G36), ranged from an average of 2992 m/s to 3260 m/s, and the differences among groups were not significant. Post-demineralization UV progressively decreased with time from G6 (2840 m/s) to G36 (2327 m/s) to approximately 92.43% of the pre-demineralization value in G6, 87.39% in G12, 78.96% in G24 and 78% in G36. Significant differences (*p*<0.0001) were found between pre- and post-demineralization intra-group values for all groups (Table 1, Figure 5). The differences among the post-demineralization values showed variable significance for comparisons made between pairs of groups (G6xG12: *p*=0.0027; G6xG24: *p*<0.0001; G6xG36: *p*<0.0001; G12xG24: *p*=0.0037; G12xG36: *p*<0.0001; G24xG36: *p*<0.0001).

### Bone mineral density

**Figure 6:**
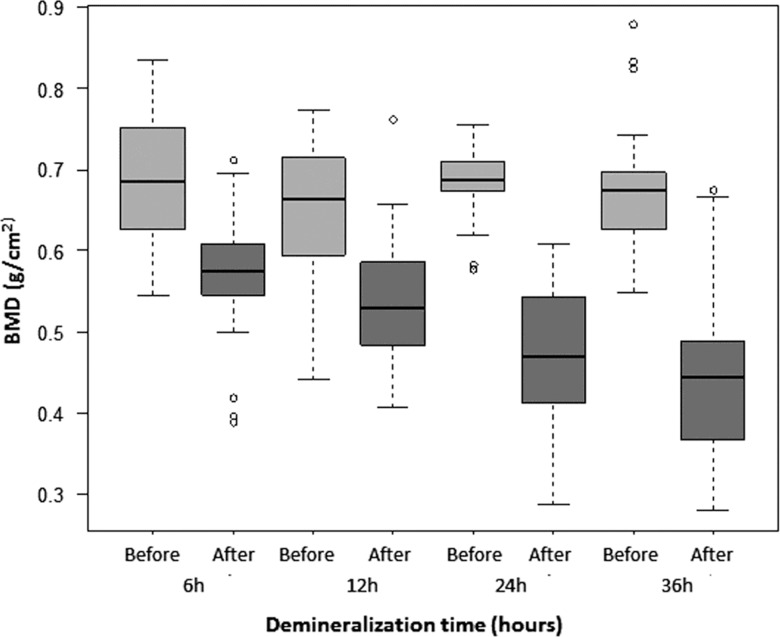
Box-plot graph of the bone mineral density (BMD) before (light grey boxes) and after (dark grey boxes) demineralization, according to demineralization time.

The mean pre-demineralization BMD ranged from 0.65 to 0.69 g/cm^2^ with no significant difference among groups. The post-demineralization BMD progressively decreased from 0.54 g/cm^2^ in G6 to 0.44 g/cm^2^ in G36, corresponding to 83.32%, 82.96%, 67.37% and 65.37% of the intra-group pre-demineralization values for G6, G12, G24 and G36, respectively, with significant differences (*p*<0.0001) between the pre- and post-demineralization values in all groups (Table 2, Figure 6). A significant difference was also observed for most comparisons of the post-demineralization values between groups (G6xG12: *p*=0.0245; G6xG24: *p*<0.0001; G6xG36: *p*<0.0001; G12xG24: *p*<0.0001; G12xG36: *p*<0.0001), but not for and G24xG36 (*p*=0.1381).

**Figure 7:**
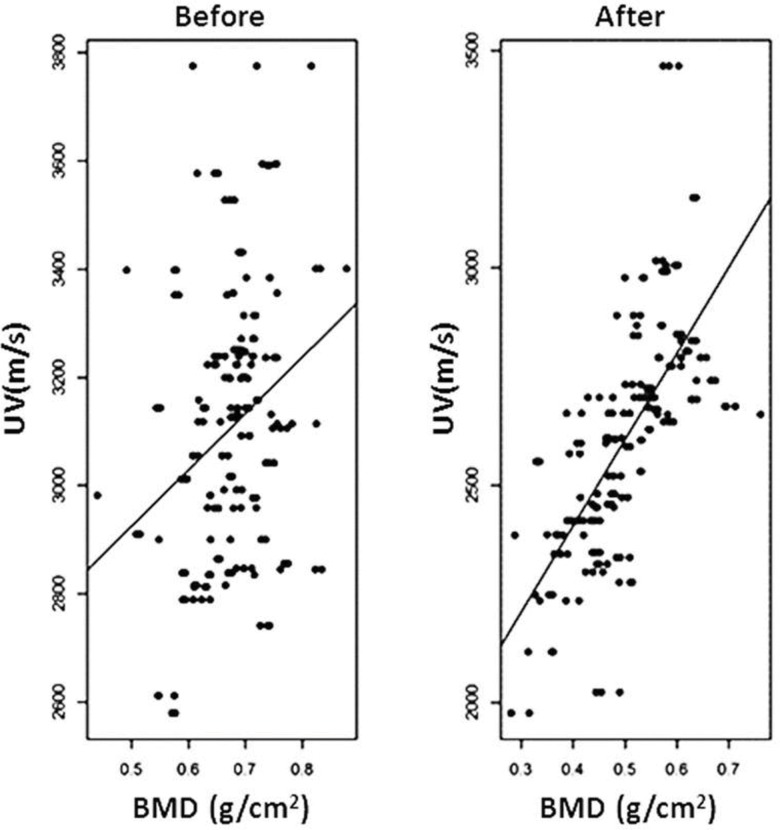
Graph of the individual UV x BMD dispersion before and after demineralization. The concentration of the dots around the average line is evident after demineralization.

The correlation between UV and BMD progressively increased with the post-demineralization period (G6: r=0.38308, *p*=0.0002; G12: r=0.64451, *p*<0.0001; G24: r=0.88733, *p*<0.0001; G36: r=0.89270, *p*<0.0001), with a relatively high overall correlation of r=0.75956 (*p*<0.0001) (Figure 7).

**Figure 8:**
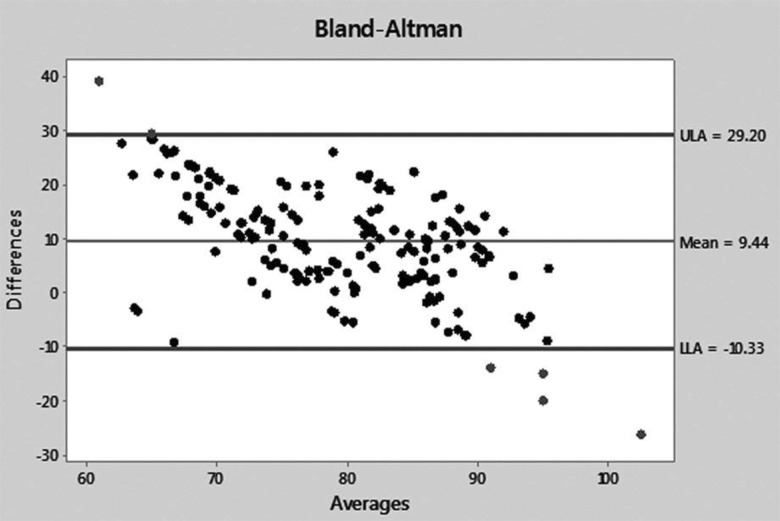
Bland-Altman graph showing the distribution of the individual average points dispersed around the mean line but concentrated between the ULA and LLA lines; a single case was above the ULA, while four were below the LLA.

UV and BMD figures were then transformed into percent values of baseline, taking the pre-demineralization values as the baseline. The differences and averages of the obtained values were then applied to a Bland-Altman graph to evaluate the agreement between the two methods. The Bland-Altman graph allows for visualization of how much each difference deviates from zero (bias), the dispersion of the differences around the average (error) and the tendency of the distribution. A perfect agreement occurs when the mean (bias) is zero and the dispersion between the upper (ULA) and lower (LLA) limits of agreement is very small or as close as possible to the mean line; data above ULA and below LLA are not in agreement. In the present case, a moderate agreement was observed because the mean was well above the zero line [9.44] and the average dots were dispersed around the mean line, although concentrated between the ULA and LLA lines. A single case was above ULA, while four cases were below LLA (Figure 8). The dot distribution also showed that lower percent differences between UV and BMD corresponded to averages closer to 100 and that this tendency of the comparison likely indicates that the agreement between the two methods is easier to detect in normal untouched bones.

## DISCUSSION

Composite materials consist of two or more materials that highly differ in character but the combination of which usually results in a third material with superior physical and mechanical properties. This is the case for bone, in which the combination of a protein matrix (collagen) and a mineral component (mostly hydroxyapatite) results in a far stronger tissue that is also flexible. However, due to the nature of its synthesis, bone is likely to show more variation in measurable physical and mechanical properties than typical engineering composites. Such variability is caused by several factors, such as age, gender, anatomical location, general health status, local disease and so on, all of which may affect the composition and structure of bone, particularly its collagen and mineral contents. Additionally, bone is an anisotropic material (with physical and mechanical properties that vary according to axle), and therefore, the measured values of a given property commonly fall within a relatively wide range; the average of the measured values is simply used as a reference value.

On clinical grounds, bone quality can be evaluated by a variety of methods, including some well-established methods, such as X-ray based methods, and some experimental methods that are still under investigation. DEXA includes exposing the patient to two low-dose x-ray beams with different energy level peaks. One peak is absorbed by the soft tissue, the energy of one peak which is then subtracted from the total, thus leaving the amount corresponding to the bone. DEXA is the current standard method for measuring BMD, which is altered in several diseases, including osteopenia and osteoporosis. Although it is a noninvasive and fast method, it involves the use of ionizing radiation, which is coupled with potential cumulative hazardous effects for patients who need repetitive examinations or also undergo other forms of examination that involve radiation.

A second alternative is ultrasonometry, or quantitative ultrasound. Although bone is an organic composite material, it has physical and mechanical properties comparable to those of man-made engineering materials (metals, plastic and so on); therefore, bone is often examined in a similar manner. Accordingly, since the 1980s, quantitative ultrasound has been investigated as a means to measure bone quality and anisotropy in an attempt to quantify osteoporosis and to estimate the risk of fracture, with the use of specific equipment 10,22,23,24. The use of the so-called transmission ultrasonometry for this purpose is based on the observation that physical properties interfere with the propagation of ultrasound waves while passing through a bone segment. However, ultrasonometry does not provide a measure of areal (g/cm^2^) bone density, such as DEXA does, but rather the UV and attenuation that can be measured while the ultrasound waves pass through a bone segment. UV and attenuation vary according to structure, density, elasticity and other physical and mechanical properties of bone 9. Therefore, theoretically, both could be used to provide an indirect measure of those properties. It has already been demonstrated, for instance, that ultrasonometry can help diagnose the healing status of fractures and can be used as an ancillary method for that purpose, particularly when the precise situation cannot be assured by conventional methods (radiographs, CT) beyond any doubt 25,26,27,28,29,30,31.

Although regularly used by many professionals to evaluate bone mass and density, ultrasonometry seems to be still an experimental method, despite the long existence of many commercially available devices 15. We believe that new clinical and/or experimental studies would be helpful in clarifying doubts regarding the ultrasonometry method and technique, particularly for cortical bone, which is less prone to both collagen and mineral content variability due to its compact structure. The aim of the present study was to determine the correlation between UV, as measured by specific equipment, and BMD, as measured by DEXA, in an experimental bench-top setup using whole cortical diaphyseal bone segments before and after demineralization. The ultrasound equipment used had already been exhaustively tested in many investigations of cortical bone, and the demineralization method was also routinely used in our laboratory.

Accordingly, we used the underwater transverse ultrasonometry modality exactly as used in our previous investigations. In this method, both emitting and receiving transducers are focused on the region of interest of the analyzed bone segment and the ultrasonic waves cross the entire bone from side to side, thus providing a more complete evaluation of the entire bone thickness and likely a more accurate measure of the UV. Actually, in the longitudinal modality, the ultrasound waves tend to run superficially, thus only evaluating the subperiosteal layer of the entry cortex depending on the US frequency/wavelength. It is well known that, for a normal (90°) ultrasound incidence, the ultrasound waves travel superficially if the wavelength is smaller than the thickness of the cortex, thus providing no information pertaining to the deep parts of the specimen. A more complete evaluation can be performed when the wavelength is greater than the thickness of the cortex, a situation in which the ultrasound waves travel through the entire cortex thickness 32. The 1 MHz frequency emission used here produced ultrasound waves of 1.5 mm wavelength, which is approximately the thickness of the cortex of the analyzed bones, therefore indicating that the emitted ultrasound waves were capable of travelling through the entire thickness.

However, the cylindrical shape of the bone shaft imposes a ring-fashion pattern to the wave propagation by which the waves running along both the anterior and posterior cortices exit on the opposite side of the emission together with a few waves that manage to pass directly through the bone marrow at a lower UV 33.

The demineralization intervals (6, 12, 24 and 36) selected in our experimental design were short because we intended to check the UV capability to detect small differences between intervals. The degree of demineralization was verified by DEXA examination at all time points (6, 12, 24 and 36 hours) of the investigation to ensure that the demineralization method was as effective as we believed and that the results of the UV and BMD measurements obtained could be compared among groups without restriction. Accordingly, the mean post-demineralization UV and BMD values progressively and significantly decreased in a linear manner with demineralization time. UV reached approximately 74% of the average pre-demineralization value and 82% of the 6-hour post-demineralization (G6) value at the 36-hour demineralization (G36) time point; BMD followed that behavior, reaching 65% and 81.5%, respectively, for the same comparisons. The intra- and inter-group differences were significant for most comparisons for both separate UV and BMD data and also between UV and BMD, indicating a moderately positive (r=0.75956) correlation coefficient and a favorable agreement between the two methods according to the Bland-Altman graph. These findings indicate that the methods are highly similar and that either method can be used to assess the loss of mineral content in cortical bone.

In conclusion, the above results clearly confirm the dependence of ultrasound wave propagation on BMD at the level of demineralization obtained during the period of study. The UV values adequately correlated and agreed with the BMD values; therefore, UV can be measured to evaluate cortical bone quality in bones affected by the loss of mineral content. However, we suggest that in clinical practice, the UV method should be limited to superficial bones such as the tibia, ulna and clavicle, as currently available equipment does not permit the examination of deeper bones surrounded by thick muscle layers.

## AUTHOR CONTRIBUTIONS

de Mesquita AQ was responsible for the experimental design, surgical and evaluation procedures, preliminary data interpretation and manuscript preparation. Barbieri G was responsible for the experimental design and for the ultrasound equipment and methodology. Barbieri CH was responsible for the experimental design, financial support (FAPESP grant), equipment provision, final data interpretation and English manuscript preparation.

## Figures and Tables

**Table 1:** UV (m/s) data (mean±SD, range, median) before and after demineralization according to demineralization time (hours).

UV	6 h	12 h	24 h	36 h
**Pre-**	3078±303	3096±226	3261±184	2988±224
	(2611 - 3776)	(2789 - 3592)	(2958 - 3595)	(2579 - 3401)
	3092	3055	3224	2992
**Post-**	2840±238	2704±188	2573±130	2327±208
	2419 - 3465	2419 - 3162	2341 - 2793	1975 - 2741
	2808	2674	2573	2319

**Table 2:** BMD (g/cm^2^) data (mean±SD, range, median) before and after demineralization according to demineralization time (hours).

BMD	6 h	12 h	24 h	36 h
**Pre-**	0.69±0.08	0.65±0.08	0.69±0.04	0.67±0.07
	(0.55 - 0.83)	(0.44 - 0.77)	(0.58 - 0.76)	(0.55 - 0.88)
	0.69	0.66	0.69	0.68
**Post-**	0.54±0.08	0.46±0.08	0.46±0.08	0.44±0.10
	(0.41 - 0.76)	(0.29 - 0.61)	(0.29 - 0.61)	(0.28 - 0.68)
	0.53	0.47	0.47	0.45
